# A Noradrenergic Lesion Attenuates Surgery-Induced Cognitive Impairment in Rats by Suppressing Neuroinflammation

**DOI:** 10.3389/fnmol.2021.752838

**Published:** 2021-11-30

**Authors:** Jiayu Wang, Ying Zhou, Ke Li, Xiaofeng Li, Meimei Guo, Mian Peng

**Affiliations:** Department of Anesthesiology, Zhongnan Hospital of Wuhan University, Wuhan, China

**Keywords:** postoperative dysfunction, locus coeruleus noradrenergic system, neuroinflammation, DSP-4, microglia

## Abstract

Postoperative cognitive dysfunction (POCD) is a common postoperative neurocognitive complication in elderly patients. However, the specific pathogenesis is unknown, and it has been demonstrated that neuroinflammation plays a key role in POCD. Recently, increasing evidence has proven that the locus coeruleus noradrenergic (LCNE) system participates in regulating neuroinflammation in some neurodegenerative disorders. We hypothesize that LCNE plays an important role in the neuroinflammation of POCD. In this study, 400 μg of N-(2-chloroethyl)-N-ethyl-2-bromobenzylamine (DSP-4) was injected intracerebroventricularly into each rat 7 days before anesthesia/surgery to deplete the locus coeruleus (LC) noradrenaline (NE). We applied a simple laparotomy and brief upper mesenteric artery clamping surgery as the rat POCD model. The open field test, novel objection and novel location (NL) recognition, and Morris water maze (MWM) were performed to assess postoperative cognition. High-performance liquid chromatography (HPLC) was used to measure the level of NE in plasma and brain tissues, and immunofluorescence staining was applied to evaluate the activation of microglia and astrocytes. We also used enzyme-linked immune-sorbent assay (ELISA) to assess the levels of inflammatory cytokines and brain-derived neurotrophic factor (BDNF). Pretreatment with DSP-4 decreased the levels of systemic and central NE, increased the level of interleukin-6 (IL-6) in the plasma at 6 h after the surgery, decreased the concentration of IL-6 in the prefrontal cortex and hippocampus, and decreased the level of interleukin-1β (IL-1β) in the plasma, prefrontal cortex, and hippocampus at 1 week postoperatively. In addition, DSP-4 treatment attenuated hippocampal-dependent learning and memory impairment in rats with POCD, with a downregulation of the activation of microglia and astrocytes in the prefrontal cortex and hippocampus. In conclusion, these findings provide evidence of the effects of LCNE in modulating neuroinflammation in rats with POCD and provide a new perspective in the prevention and treatment of POCD.

## Introduction

Postoperative cognitive dysfunction (POCD) is one of the most common postoperative complications in elderly patients (Granger and Barnett, 2021) and is characterized by declines in learning, memory, attention, and executive capability following anesthesia and surgery (Chen et al., 2019; Yan et al., 2020). Clinical evidence indicates that POCD may contribute to increased mortality, decreased quality of life, prolonged hospitalization, and increased burden on the medical care system (Steinmetz et al., 2009). However, to date, the neuropathogenesis of POCD remains unknown.

Neuroinflammation has been demonstrated to play a vital role in the occurrence and development of POCD (Soriano et al., 2017; Subramaniyan and Terrando, 2019; Yang et al., 2019). Surgical trauma stimulates the innate immune system, leading to the release of proinflammatory cytokines, such as tumor necrosis factor-α (TNF-α), interleukin-6 (IL-6), and interleukin-1β (IL-1β), in the systemic circulation (Hirsch et al., 2016), which ultimately compromises the blood–brain barrier (BBB) and promotes monocyte-derived migration of macrophages into the brain parenchyma (Lv et al., 2010), resulting in the activation of microglia and astrocytes (Xu et al., 2017; Hu et al., 2018). Overactivated microglia elicit detrimental effects by the overexpression of cytokines, and reactive astrocytes acquire toxic functions and lose neurotrophic functions (Kaur et al., 2019). The interaction between peripheral and central immune responses aggravates neuroinflammation and neuronal damage, which ultimately impairs cognitive function (Terrando et al., 2011; Alam et al., 2018).

The locus coeruleus (LC) is the main noradrenergic nucleus of the brain and releases the neurotransmitter norepinephrine in many anatomically and functionally diverse brain regions (Schwarz and Luo, 2015; Benarroch, 2018). The locus coeruleus noradrenergic (LCNE) pathway has been proven to be involved in regulating a wide range of advanced cognitive functions (Sara, 2009; Betts et al., 2019), such as working memory, learning and attention (Robbins, 1984; Aston-Jones and Cohen, 2005; Mather et al., 2016), memory formation and consolidation (Gibbs and Summers, 2002; Gibbs et al., 2010; Hansen, 2017), and immunological mechanisms in the brain (Polak et al., 2011; Stowell et al., 2019). A few studies have shown that the LCNE pathway contributes to the pathomechanism of neurodegenerative diseases, such as Alzheimer’s disease (AD), Parkinson’s disease (PD) (Giorgi et al., 2020), and septic encephalopathy (O’Neill et al., 2020), in which neuroinflammation plays an important role. In Termpanit’s study, the losses of LC fibers and noradrenaline (NE) exacerbate spatial learning and memory deficits in transgenic mouse models of AD, accompanied by the activation of microglia and astrocytes in the hippocampus (Chalermpalanupap et al., 2018). In primary rat cortical microglial cells, exogenous NE decreased lipopolysaccharide- (LPS-) induced microglial nitric oxide synthase (NOS2) expression and IL-1β production, which was mediated by a β_2_ adrenergic receptor (β_2_-AR) (Dello Russo et al., 2004).

To date, the effects of the LCNE system on the neuroinflammatory mechanism of POCD have not been reported. Based on the crucial role of LCNE in neuroinflammation-related neurodegenerative diseases, we hypothesize that the LCNE system contributes to the neuroinflammation of rats with POCD.

N-(2-Chloroethyl)-N-ethyl-2-bromobenzylamine (DSP-4) is a competitive inhibitor of NE uptake and selectively degenerates noradrenergic neurons originating from the LC, whereas noncerulean-innervated noradrenergic axons are unaffected (Fritschy and Grzanna, 1991; Kudo et al., 2011). Thus, DSP-4 has been widely used as a noradrenergic neurotoxin. In this study, we adopted intracerebroventricular administration of DSP-4 at 400 μg per rat (Archer et al., 1984; Chan et al., 1991) to investigate the effects of LCNE lesions on neuroinflammation and cognitive function in POCD model rats.

## Materials and Methods

### Animals and Groups

All procedures were approved by the Animal Ethics Committee of Zhongnan Hospital of Wuhan University, Hubei, China, and all experiments were performed in accordance with the National Institutes of Health Guidelines for the Care and Use of Laboratory Animals. The ethical number of Animal Using Protocol (AUP) is WP202008006. Efforts were made to minimize the number of animals used. Wistar rats (12 weeks old, male, weighing 250–350 g) were purchased from SPF (Beijing) Biotechnology Co. Ltd. (Beijing, China). All animals were group-housed four per cage with free access to food and water. The temperature, humidity, and day–night cycle were maintained according to the standards established by the experimental animal laboratory at Zhongnan Hospital of Wuhan University. The rats were allowed 1 week to acclimatize to the laboratory environment before the experiment.

Rats were randomly divided into four groups: a vehicle (V) group (*n* = 10), a DSP-4 (D) group (*n* = 10), a vehicle + surgery (V + S) group (*n* = 18), and a DSP-4 + surgery (D + S) group (*n* = 18).

### Surgery

Rats in Groups V + S and D + S received a simple laparotomy and brief upper mesenteric artery clamping surgery (Hovens et al., 2013, 2014). Specifically, each rat was induced with 3% sevoflurane in 100% oxygen in a transparent acrylic chamber. Fifteen minutes after induction, the rats were removed from the chamber and placed on a heating pad to keep their body temperature between 37 and 38°C. Sevoflurane anesthesia was maintained *via* a cone device, and a 16-gauge needle was inserted into the cone near the nose of the rat to monitor the concentration of sevoflurane. A longitudinal midline incision of approximately 3 cm was made on the skin, abdominal muscles, and peritoneum. Then, the gastrointestinal artery was exteriorized, and the upper mesenteric artery was clamped for 30 min. Clamping the upper mesenteric artery results in a restricted flow to the mesenteric vascular bed although the presence of collateral arteries allows some perfusion (Hovens et al., 2014). Then, the incision was sutured layer-by-layer with 3–0 Vicryl thread, and the rats were put back into an anesthesia chamber for up to 1 h. A heat pad was used to keep their body temperature between 36 and 37°C during the surgery. After recovering from anesthesia, the rats were returned to their home cage with food and water available. The rats in Groups V and D did not receive anesthesia or surgery. All rats with surgery received postsurgical analgesia (flunazine 2.5 mg/kg daily) for 48 h.

### Drug Preparation and Injection

N-(2-chloroethyl)-N-ethyl-2-bromobenzylamine (neurotoxin DSP-4 hydrochloride, MCE, HY-103210) was given at 400 μg/rat and dissolved in sterile saline 0.9%. Rats were anesthetized with chloral hydrate (300 mg/kg, i.p.). DSP-4 was injected slowly in a volume of 10 μl into the right lateral ventricle (rostral-caudal: −0.8 mm relative to bregma; medial-lateral: −1.8 mm; dorsal-ventral: −3.6 mm from the skull) 7 days before the surgery, and the coordinate was based on The Rat Brain in Stereotaxic Coordinates, the sixth edition of Elsevier Press. The rats in Groups V and V + S were injected with the same volume of sterile saline (0.9%).

### Behavioral Tests

**FIGURE 1:**
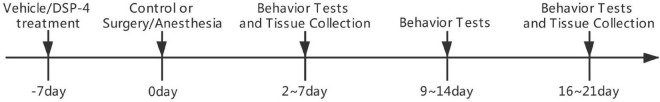
Diagram of the experimental design. Groups V+S and D+S were operated on 7 days after intracerebroventricular injection. Behavioral tests 1, 2, and 3 weeks after the surgery, including a series of tests, started on days 2, 9, or 16 and lasted for 5 days. Rats were sacrificed 1 day after the last behavioral tests in the 1st and 3rd week after the surgery.

Behavioral tests included open field tests, novel objection and novel location (NL) recognition, and Morris water maze (MWM) tests at 1, 2, and 3 weeks postoperatively (Figure 1) (Hovens et al., 2014).

#### Open Field

The open field test was performed to assess motor function and exploratory function. The test was performed 2, 9, and 16 days after anesthesia and surgery. Specifically, the rat was placed in the center of an open field chamber (100 cm × 100 cm × 100 cm) under dim light and was allowed to move freely for 5 min, and the field was divided into a center area (60 cm × 60 cm), four corner areas (20 cm × 20 cm), and four side areas (20 cm × 60 cm). The activities were automatically recorded by a video camera connected to the Any-Maze animal tracking system software (Xinruan Information Technology Co. Ltd., Shanghai, China), and movement parameters were calculated by the software. The total distance moved was recorded and analyzed.

#### Novel Objection and Novel Location Recognition

The novel objection and novel location recognition test was performed to assess visual and spatial short memory (Wang et al., 2016). The test was performed 3, 10, and 17 days after anesthesia and surgery. Specifically, the day before testing, rats were placed into the testing box (50 cm ×50 cm × 40 cm) two times to acclimate for 5 min. On the testing day, the test consisted of three phases of 3 min, separated by a 45-s pause, and the rat remained in the testing box. In the exploration phase, the rat was presented with two identical objects (plastic bottles filled with water). In the novel object (NO) recognition phase, the rat was presented with a familiar object and a NO. In the novel location (NL) recognition phase, the familiar object from NO did not change its location, while the novel object from NO was placed in a NL. All apparatuses were cleaned with 75% alcohol to remove odors. The time spent exploring objects was recorded by a video camera connected to the Any-Maze animal tracking system software (Xinruan Information Technology Co. Ltd., Shanghai, China), and movement parameters were calculated by the software. The ratio of time the animal spent exploring the novel or relocated object compared to the total object exploration time was considered as a measure of object or location recognition. Trials in which the rats spent less than 5 s exploring the objects were removed from further analysis.

#### Morris Water Maze

The MWM test was performed to assess spatial learning, spatial memory, and cognitive flexibility. The MWM consists of a circular pool with a diameter of 1.5 m at a depth of 45 cm. The tank was divided into four equal quadrants, with the platform (10 cm × 10 cm) located in the center of the target quadrant. The platform was hidden 1 cm below the water surface, and black ink was used to make the water opaque. The testing room was illuminated with constant light source intensity during the experiments, and different figures and objects that could create visual clues for the rats were hung on the walls. Every rat’s swimming track was recorded by a camera above the maze. Each rat was placed randomly in the pool.

The water maze protocol started 4, 11, and 18 days postoperatively, consisting of two training phases, two testing phases, and reversal training over a period of 3 days.

On the 1st day of the protocol, the rats were trained to find the hidden platform. The first training phase included three training sessions with an interval of 1 h, and a training session consisted of three continuous trials. The rat was sequentially placed in each quadrant without a platform and allowed to search for the platform. The latency time was recorded as 60 s if the rat failed to find the platform. Each rat was allowed to stand on the platform, and the surroundings were observed for 10 s. The average escape latency of each training session was considered as a measure to assess spatial learning capacity.

On the 2nd day, the protocol was performed to assess spatial memory capacity, including a testing phase and two training sessions. In the testing phase, the platform was removed, and the rat was placed in a random quadrant and allowed to explore the maze for 60 s. The swimming paths, time spent in each quadrant, and distance moved were recorded by a camera above the maze. Time spent in the target quadrant was considered to measure spatial memory. One hour later, the rats underwent two training sessions to ensure that all the rats learned the location of a platform and had a base as the second testing and reversal training session on the 3rd day. The average escape latency of each training session was considered as a measure to assess spatial learning. We used the average escape latency of five training sessions to draw a learning curve.

The 3rd day of the protocol included the second testing phase and four continuous reversal tests. The second testing phase was the same as the previous day and was performed to assess spatial memory. The reversal tests were performed 1 h later, in which the platform was moved to an opposite quadrant. The average escape latency was taken to measure cognitive flexibility.

### Tissue Harvest

The rats were anesthetized and euthanized immediately or 7 or 21 days postoperatively, and the hippocampus and prefrontal cortex were harvested and stored at −80°C for future use. We collected 3 ml of whole blood by cardiac puncture under anesthetic. Blood was centrifuged at 2,500× *g* for 10 min at 4°C, and the plasma was collected at −80°C for further use.

### High-Performance Liquid Chromatography

High-performance liquid chromatography (HPLC) was conducted on plasma and prefrontal cortex and hippocampal tissue homogenates as discussed in previous publications (Finnell et al., 2019) 7 days after the operation. Briefly, all samples were spiked with 1 ng/ml 3,4-dihydroxybenzylamine hydrobromide (DHBA, Sigma, St. Louis, MO, United States, 858781) to serve as an internal standard, mixed with perchloric acid (4 mol/L), and centrifuged at 1,500× *g* for 15 min at 4°C. Supernatant was collected, in which 30 μl of pickled alumina and 1.5 ml of Tris-HCL (1.5 mol/L, pH 8.6, 1% EDTA-Na_2_) were added, and the sample was centrifuged at 1,500× *g* for 15 min at 4°C. The supernatant was discarded, the pellet was washed three times with water, the supernatant was collected, and the sample was shocked with 0.1 mol/L perchloric acid and centrifuged at 1,500× *g* for 15 min at 4°C. One hundred microliters of supernatant were used for a HPLC analysis, and all NE data were normalized to DHBA. According to the peak area and standard concentration, the content of norepinephrine in the samples was calculated by the recovery of the internal standard.

### Enzyme-Linked Immune-Sorbent Assay

Rat ELISA kits to detect IL-6 (ELK Biotechnology, Wuhan, China, ELK1158), IL-1β (ELK Biotechnology, Wuhan, China, ELK1272), and brain-derived neurotrophic factor (BDNF) (ELK Biotechnology, Wuhan, China, ELK5459) were used to evaluate the concentrations of IL-6, IL-1β, and BDNF in the plasma, prefrontal cortex, and hippocampus.

### Immunofluorescence

Seven days after the surgery, each rat was anesthetized with 1.4% isoflurane and perfused transcardially with ice-cold 0.1 M PBS followed by 4% PFA in 0.1 M PBS at pH 7.4. Brains were harvested and fixed in 4% PFA in 0.1 M PBS at 4°C, cryoprotected in 30% sucrose for 72 h, frozen in TissueTek OCT (Sakura), and cut sequentially to 20 μm. After washing in PBS and permeabilization in 0.5% Triton X-100, the sections were blocked with 10% goat serum for 2 h at room temperature to block nonspecific binding and were washed in PBS. Then, the sections were incubated with rabbit anti-Iba-1 (1:200, Abcam, Cambridge, United Kingdom, ab178847) or mouse anti-glial fibrillary acidic protein (GFAP) (1:500, Invitrogen, Waltham, MA, United States, MA5-12023) primary antibodies at 4°C overnight. After washing, the sections were incubated with secondary antibody (goat anti-rabbit) conjugated with CY3 (1:400) or secondary antibody (goat anti-mouse) conjugated with Alexa Fluor dye 488 (1:200) from Invitrogen at room temperature for 2 h in the dark. Immunolabeled sections were coverslipped with 40,6-diamidino-2-phenylindole (DAPI; Invitrogen, Waltham, MA, United States) and analyzed by microscopy (Olympus, Tokyo, Japan) equipped with an imaging system. Five high magnifications were chosen in three nonoverlapping fields randomly acquired in hippocampal and prefrontal cortex subregions using a counting frame size of 0.4 mm^2^. Images were processed, and the area of the microglia was quantified using the ImageJ software (NIH). The area of the selected cells was converted into immunoreactivity, which was calculated as the percentage area density defined as the number of pixels (positively stained area) divided by the total number of pixels (the sum of positively and negatively stained area) in the imaged field.

### Statistical Analysis

Statistical analysis was performed with SPSS 23.0 (IBM, New York, NY, United States) or GraphPad Prism 6 (GraphPad, New York, NY, United States). Quantitative data are expressed as the means ± SEM. Statistical significance was determined using one-way or two-way ANOVA followed by Tukey’s *post hoc* multiple comparison tests. The value of *p* < 0.05 was considered statistically significant.

## Results

### Effects of ICV Injection of DSP-4 on the Level of Noradrenaline

**FIGURE 2:**
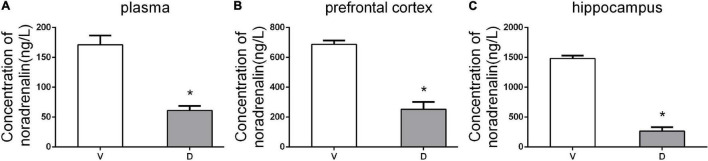
DSP-4 treatment decreased the concentrations of noradrenaline (NE) in the plasma, prefrontal cortex, and hippocampus 7 days postoperatively. **(A)** NE concentrations (ng/L) in plasma. **(B)** NE concentrations (ng/L) in the prefrontal cortex. **(C)** NE concentrations (ng/L) in the hippocampus. The data are presented as the means ± SEM for each group (*n* = 5 per cohort). *p < 0.05 compared to Group V.

To confirm the effect of ICV administration of DSP-4, HPLC detection was conducted to measure the levels of NE in the plasma, prefrontal cortex, and hippocampus 7 days after the operation. HPLC results showed that DSP-4 injection decreased NE by 63.15% in the prefrontal cortex (average ± SEM (ng/L): vehicle: 686.75 ± 13.14; DSP-4: 253 ± 24.45), 82.07% in the hippocampus (average ± SEM (ng/L): vehicle: 1,482.25 ± 23.73; DSP-4: 265.75 ± 31.69), and 64.32% in the plasma (average ± SEM (ng/L): vehicle: 171 ± 7.78; DSP-4: 61 ± 3.85) (*p < 0.05*, Figure 2). These results confirm that DSP-4 treatment successfully depleted NE in the hippocampus and frontal cortex.

### Effects of ICV Injection of DSP-4 on Motor Function and Nonhippocampal-Dependent Memory

**FIGURE 3:**
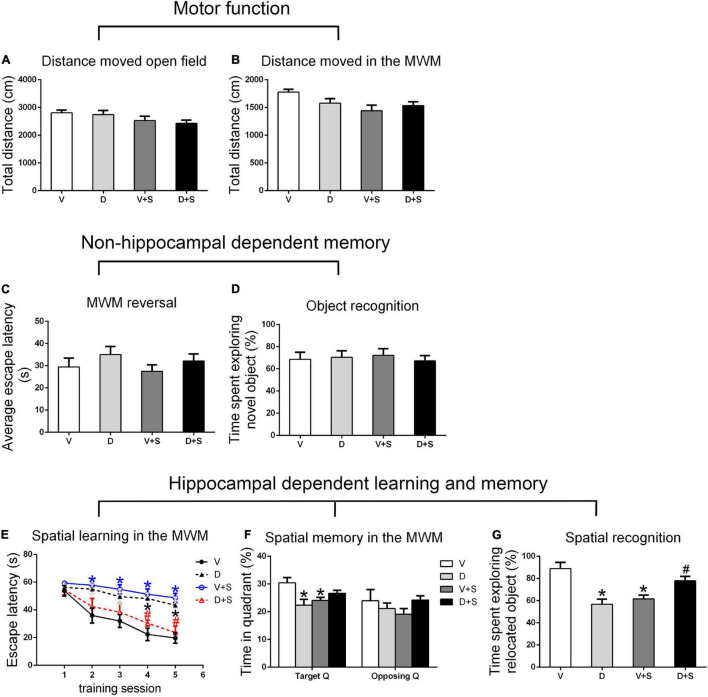
Open field behavior, learning, and memory performance in the four groups 1 week postoperatively. **(A)** Distance moved in the open field test (cm). **(B)** Distance moved (cm) in the Morris water maze (MWM) during the first test as a measure of motor function. **(C)** Cognitive flexibility in the MWM. Average escape latency (s) during the four MWM reversal trials. **(D)** Object recognition in the novel object (NO) test. **(E)** Spatial learning in the MWM. Average escape latency (s) is shown for the five training sessions in the maze. **(F)** Spatial memory in the MWM. The percentages of time in the target quadrant (Target Q) and time in the quadrant opposite to the target quadrant (Opposing Q) are shown. **(G)** Spatial recognition in the novel location (NL) test. Exploration time of the relocated object as a percentage of total object exploration is shown. The data are presented as the means ± SEM for each group (*n* = 10 per cohort). *p < 0.05 compared to Group V, ^#^*p* < 0.05 compared to Group V + S.

**FIGURE 4:**
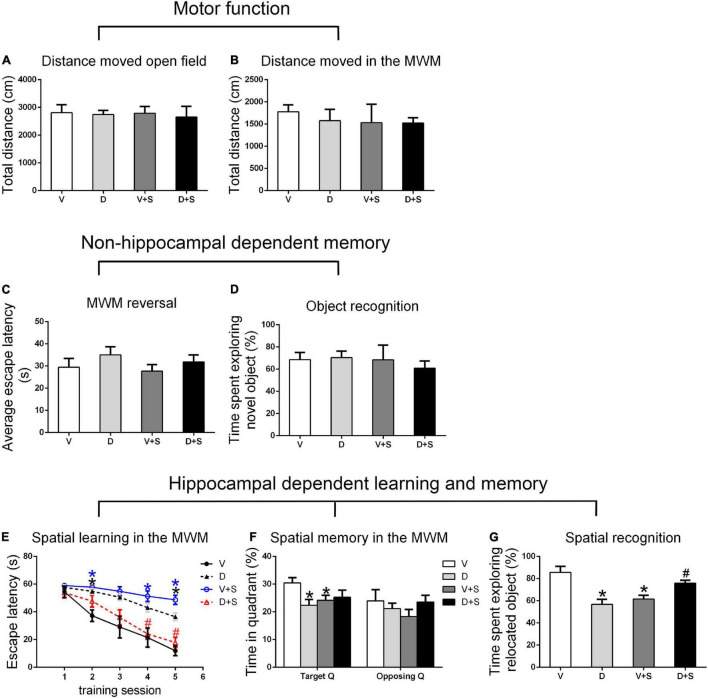
Open field behavior, learning, and memory performance in the four groups at 2 weeks postoperatively. **(A)** Distance moved in the open field test (cm). **(B)** Distance moved (cm) in the MWM during the first test as a measure of motor function. **(C)** Cognitive flexibility in the MWM. Average escape latency (s) during the four MWM reversal trials. **(D)** Object recognition in the NO test. **(E)** Spatial learning in the MWM. Average escape latency (s) is shown for the five training sessions in the maze. **(F)** Spatial memory in the MWM. The percentages of time in the target quadrant (Target Q) and time in a quadrant opposite to the target quadrant (Opposing Q) are shown. **(G)** Spatial recognition in the NL test. Exploration time of the relocated object as a percentage of total object exploration is shown. The data are presented as the means ± SEM for each group (*n* = 10 per cohort). *p < 0.05 compared to Group V, ^#^*p* < 0.05 compared to Group V + S.

**FIGURE 5:**
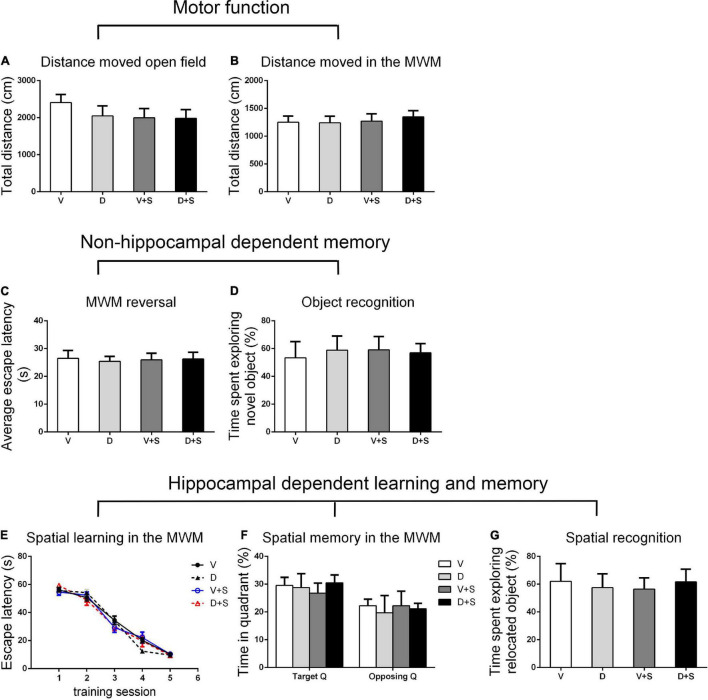
Open field behavior, learning, and memory performance in the four groups at 3 weeks postoperatively. **(A)** Distance moved in the open field test (cm). **(B)** Distance moved (cm) in the MWM during the first test as a measure of motor function. **(C)** Cognitive flexibility in the MWM. Average escape latency (s) during the four MWM reversal trials. **(D)** Object recognition in the NO test. **(E)** Spatial learning in the MWM. Average escape latency (s) is shown for the five training sessions in the maze. **(F)** Spatial memory in the MWM. The percentages of time in the target quadrant (Target Q) and time in the quadrant opposite to the target quadrant (Opposing Q) are shown. **(G)** Spatial recognition in the NL test. Exploration time of the relocated object as a percentage of total object exploration is shown. The data are presented as the means ± SEM for each group (*n* = 10 per cohort). *p < 0.05 compared to Group V, ^#^*p* < 0.05 compared to Group V + S.

There were no significant differences in the distance moved in the open field test and MWM among the four groups (*p > 0.05*, Figures 3A,B, 4A,B, 5A,B) at 1, 2, and 3 weeks postoperatively, indicating that surgery or DSP-4 treatment does not affect motor function in rats.

No significant differences were found in escape latency during the MWM reversal trials (*p > 0.05*, Figures 3C, 4C, 5C) or the time spent exploring NOs during the NO phase (*p > 0.05*, Figures 3D, 4D, 5D), which suggested that nonhippocampal-dependent memory was not influenced by surgery or NE lesions.

### Effects of ICV Injection of DSP-4 on Hippocampal-Dependent Learning and Memory

Hippocampal-dependent learning and memory included training sessions in the MWM, time spent in the target quadrant during the testing phase in the MWM, and time spent exploring the relocated objects during the NL phase. To determine the effect of surgery, DSP-4 treatment, and the interactions on cognitive impairment, we used two-way ANOVA and Tukey’s *post hoc* multiple comparison tests to analyze behavioral changes.

At 1 and 2 weeks postoperatively, the learning curve (Figures 3E, 4E) of escape latency in the training session showed an obvious downward tendency, indicating that all rats were able to learn where the platform was located. The escape latency in Group V + S was markedly elevated compared with that in Group V, and the pretreatment with DSP-4 diminished the learning impairment caused by surgery. The learning curve showed that training four was the time point with the most obvious difference among the four groups. A two-way ANOVA on the escape latency of training four showed significant effects of the surgery (1 week postoperative: *F*_1,38_ = 3.165, *p = 0.084*; 2 weeks postoperative: *F*_1,32_ = 10.974, *p = 0.002*) as well as the interaction between surgery and DSP-4 treatment (1 week postoperative: *F*_1,38_ = 30.333, *p < 0.001*; 2 weeks postoperative: *F*_1,32_ = 7.307, *p = 0.011*). Subsequent Tukey’s *post hoc* analyses indicated that DSP-4 treatment (1 week postoperative: *p = 0.02*; 2 weeks postoperative: *p = 0.001*, Figures 3E, 4E) improves the learning impairment caused by surgery.

According to the result of a two-way ANOVA in time spent in the target quadrant, the interaction of surgery and DSP-4 treatment had a noticeable impact on time spent in target Q at 1 week postoperatively (*F*_1,38_ = 14.727, *p* < *0.001*). At 2 weeks after the surgery, surgery (*F*_1,32_ = 4.672, *p = 0.039*) and DSP-4 treatment (*F*_1,32_ = 11.181, *p = 0.002*) both had significant effects. Tukey’s *post hoc* analyses showed that compared to Group V, both surgery (1 week postoperative: *p = 0.02*, 2 weeks postoperative: *p = 0.031*, Figures 3F, 4F) and DSP-4 treatment (1 week postoperative: *p = 0.022*, 2 weeks postoperative: *p = 0.007*, Figures 3F, 4F) led to a significantly decreased duration in the target quadrant.

In the NL phase, a two-way ANOVA showed that the interaction of surgery and treatment was the main influencing factor at 1 week postoperatively (*F*_1,38_ = 29.982, *p* < *0.001*). Then, we measured Tukey’s *post hoc* multiple comparison tests, and the results showed that location recognition was decreased in Groups D and V + S compared to Group V, while the recognition of Group D + S was improved compared to Group V + S (1 week postoperative: *p = 0.032*, 2 weeks postoperative: *p = 0.023*, Figures 3G, 4G).

At 3 weeks postoperatively, there was no significant difference in hippocampal-dependent learning and memory among the four groups (*p > 0.05*, Figures 5E–G).

In conclusion, intracerebroventricular injection of DSP-4 attenuated the impairment of hippocampal-dependent learning and memory induced by surgery in rats.

### Effects of ICV Injection of DSP-4 on Peripheral and Central Inflammatory Cytokines After Surgery

**FIGURE 6:**
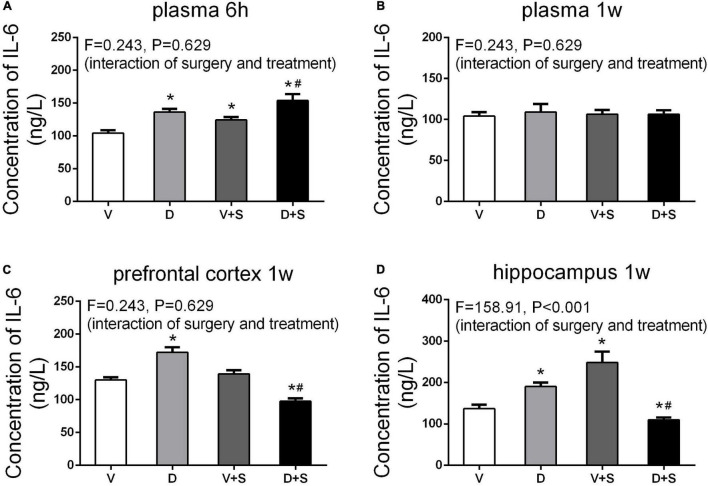
Peripheral and central concentrations of interleukin-6 (IL-6). DSP-4 treatment increased the level of plasma IL-6 under physiological and pathological conditions at 6 h after the surgery **(A)**, while the concentration of IL-6 showed no significant change at 1 week after the surgery **(B)**. At 1 week postoperatively, DSP-4 treatment increased the concentration of IL-6 in the prefrontal cortex **(C)** and hippocampus **(D)** under physiological conditions and decreased it under pathological conditions. The data are presented as the means ± SEM for each group (*n* = 4 per cohort). *p < 0.05 compared to Group V, ^#^*p* < 0.05 compared to Group V + S.

To assess the effects of DSP-4 on systemic inflammation and neuroinflammation, we measured the concentrations of IL-6 and IL-1β in the plasma, prefrontal cortex, and hippocampus after the surgery. Considering a rapid change in plasma IL-6 (Hovens et al., 2014), we measured plasma IL-6 at 6 h and 1 week postoperatively. At 6 h after the surgery, a two-way ANOVA on the levels of plasma IL-6 showed significant effects of DSP-4 treatment (*F*_1,19_ = 116.025, *p* < *0.001*) and operation (*F*_1,19_ = 43.923, *p* < *0.001*), but there was no significant interaction between treatment and surgery (*F*_1,19_ = 0.243, *p = 0.629*). Tukey’s *post hoc* analysis revealed that both DSP-4 (*p* < *0.001*, Figure 6A) and surgery (*p = 0.002*, Figure 6A) increased the level of IL-6 in plasma at 6 h after the operation. At 1 week postoperatively, there was no significant difference in plasma IL-6 among the four groups (*p > 0.05*, Figure 6B).

At 7 days after the operation, the results of a two-way ANOVA on IL-6 in the prefrontal cortex showed significant effects of the surgery (*F*_1,15_ = 132.18, *p* < *0.001*) and the interaction between treatment and surgery (*F*_1,15_ = 218.878, *p* < *0.001*). In the hippocampus, the results of two-way ANOVA showed significant effects of DSP-4 treatment (*F*_1,15_ = 31.23, *p* < *0.001*), as well as the interaction between treatment and surgery (*F*_1,15_ = 31.23, *p* < *0.001*). Subsequent Tukey’s *post hoc* test showed that 7 days after the operation, the levels of IL-6 in Group D (prefrontal cortex: *p = 0.002*, hippocampus: *p = 0.002*, Figures 6C,D) were significantly increased compared to those in Group V, and DSP-4 treatment downregulated the elevation of the surgery results (prefrontal cortex: *p* < *0.001*, hippocampus: *p = 0.009*, Figures 6C,D).

**FIGURE 7:**
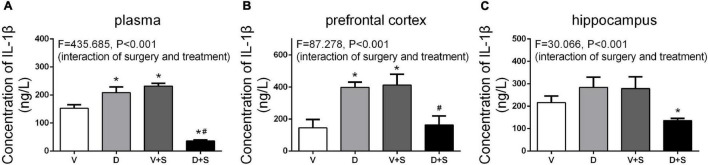
DSP-4 treatment inhibits the increase in interleukin-1β (IL-1β) levels caused by surgery at 1 week postoperatively. Prior to the central administration of DSP-4 attenuated the surgery-induced increase in IL-1β levels in the plasma **(A)**, prefrontal cortex **(B)**, and hippocampus **(C)**. The data are presented as the means ± SEM for each group (*n* = 4 per cohort). *p < 0.05 compared to Group V, ^#^*p* < 0.05 compared to Group V + S, DP < 0.05 compared to Group D.

The levels of IL-1β in the plasma, prefrontal cortex, and hippocampus were measured 1 week after the surgery. According to the results of two-way ANOVA, in the plasma, DSP-4 treatment (*F*_1,18_ = 135.154, *p* < *0.001*) and surgery (*F*_1,18_ = 59.579, *p* < *0.001*) affected the level of IL-1β, as well as the interaction between treatment and surgery (*F*_1,18_ = 435.685, *p* < *0.001*). In the prefrontal cortex, the results of two-way ANOVA showed significant effects of the interaction between treatment and surgery (*F*_1,15_ = 87.278, *p* < *0.001).* In the hippocampus, surgery (*F*_1,15_ = 4.886, *p = 0.047*) and the interaction between treatment and surgery (*F*_1,15_ = 30.066, *p* < *0.001*) affected the level of IL-1β. Tukey’s *post hoc* test indicated that surgery significantly increased IL-1β levels in the plasma (*p* < *0.001*, Figure 7A) and prefrontal cortex (*p = 0.006*, Figure 7B). In the hippocampus, there was an increasing tendency but no significant difference (*p = 0.455*, Figure 7C). Compared to Group V, the IL-1β level of Group D was upregulated in plasma (*p* < *0.001*, Figure 7A) and the prefrontal cortex (*p = 0.002*, Figure 7B), and in the hippocampus, there was an increasing tendency but not a significant difference (*p = 0.295*, Figure 7C). The concentration of IL-1β in Group D + S decreased significantly compared to Group V + S in plasma (*p* < *0.001*, Figure 7A), the prefrontal cortex (*p = 0.009*, Figure 7B), and the hippocampus (*p = 0.001*, Figure 7C).

### Effects of ICV Injection of DSP-4 on the Reactive States of Astrocytes and Microglia in the Hippocampus and Prefrontal Cortex Elicited by Surgery/Anesthesia

We measured the changes in the immunoreactivity of Iba-1 and GFAP in the prefrontal cortex and hippocampus to assess the reactive states of microglia and astrocytes, which represent the major pathological manifestation of neuroinflammation (Norden et al., 2016; Xu et al., 2017; Long et al., 2020).

**FIGURE 8:**
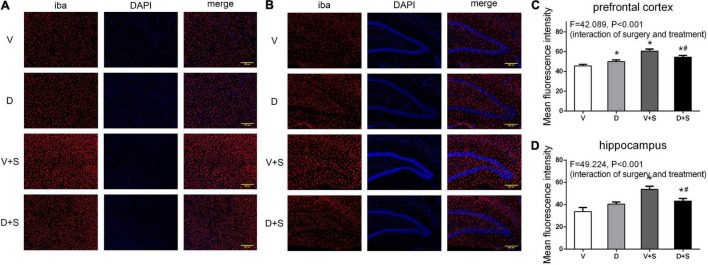
DSP-4 treatment decreased the activation of microglia in surgical rats. The activation of microglia in the prefrontal cortex **(A)** and hippocampus **(B)** at 1 week postoperatively. **(C,D)** Mean fluorescence intensity in the prefrontal cortex and hippocampus at 1 week postoperatively. The data are presented as the means ± SEM for each group (*n* = 3 per cohort). *Compared to Group V, ^#^*p* < 0.05 compared to Group V + S.

A two-way ANOVA on Iba-1 densitometry measurements showed significant effects of the surgery (prefrontal cortex: *F*_1,19_ = 146.337, *p* < *0.001*, hippocampus: *F*_1,19_ = 86.75, *p* < *0.001*), as well as the interaction between treatment and surgery (prefrontal cortex: *F*_1,19_ = 42.089, *p* < *0.001*, hippocampus: *F*_1,19_ = 49.224, *p* < *0.001*). Subsequent Tukey’s *post hoc* test showed that surgery increased Iba-1 immunoreactivity in the prefrontal cortex (*p* < *0.001*, Figures 8A,C) and hippocampus (*p* < *0.001*, Figures 8B,D) compared with Group V, while DSP-4 treatment significantly weakened staining with Iba-1 antibody compared to Group V + S (prefrontal cortex: *p = 0.007*, hippocampus: *p = 0.001*, Figure 8). Rats in Group D had increased Iba-1 immunoreactivity in the prefrontal cortex compared to that in Group V (*p = 0.021*, Figures 8A,C), but a similar phenomenon was not observed in the hippocampus (*p = 0.066*, Figures 8B,D).

**FIGURE 9:**
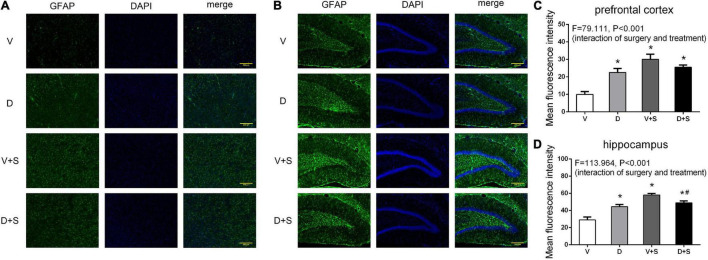
DSP-4 treatment decreased the activation of astrocytes in surgical rats. The activation of astrocytes in the prefrontal cortex **(A)** and hippocampus **(B)** at 1 week postoperatively. **(C,D)** Mean fluorescence intensity in the prefrontal cortex and hippocampus at 1 week postoperatively. The data are presented as the means ± SEM for each group (*n* = 3 per cohort). *p < 0.05 compared to Group V, ^#^*p* < 0.05 compared to Group V + S, DP < 0.05 compared to Group D.

The pretreatment with DSP-4 also attenuated the activation of astrocytes. Two-way ANOVA of GFAP densitometry measurements showed significant effects of DSP-4 treatment (prefrontal cortex: *F*_1,19_ = 17.508, *p = 0.001*, hippocampus: *F*_1,19_ = 7.520, *p = 0.014*) and surgery (prefrontal cortex: *F*_1,19_ = 144.457, *p* < *0.001*, hippocampus: *F*_1,19_ = 208.608, *p* < *0.001*), as well as the interaction between treatment and surgery (prefrontal cortex: *F*_1,19_ = 79.111, *p* < *0.001*, hippocampus: *F*_1,19_ = 113.964, *p* < *0.001*). Tukey’s *post hoc* test revealed that surgery increased GFAP immunoreactivity in the prefrontal cortex (*p* < *0.001*, Figures 9A,C) and hippocampus (*p* < *0.001*, Figures 9B,D). DSP-4 treatment decreased the activation of astrocytes in the hippocampus caused by surgery (*p = 0.001*, Figures 9B,D); however, in the prefrontal cortex, there was no significant effect on GFAP immunoreactivity between Groups V + S and D + S (*p = 0.126*, Figures 9A,C). There was enhanced GFAP immunoreactivity in Group D compared with Group V (prefrontal cortex: *p* < *0.001*, hippocampus: *p* < *0.001*, Figure 9).

### Effects of ICV Injection of DSP-4 on Brain-Derived Neurotrophic Factor Levels in the Prefrontal Cortex and Hippocampus

**FIGURE 10:**
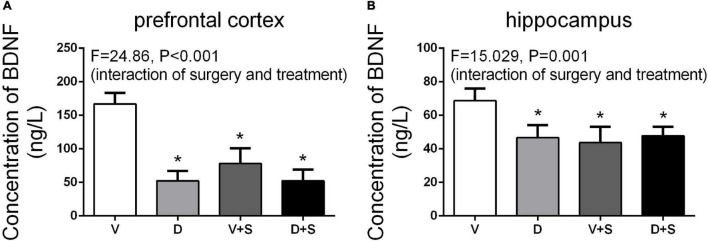
Brain-derived neurotrophic factor (BDNF) levels in the prefrontal cortex **(A)** and hippocampus **(B)** at 3 weeks postoperatively. The data are presented as the means ± SEM for each group (*n* = 5 per cohort). *p < 0.05 compared to Group V, ^#^*p* < 0.05 compared to Group V + S.

Referencing previous research (Hovens et al., 2014), we selected the time point of 3 weeks postoperatively to measure the concentration of BDNF. The results of two-way ANOVA on BDNF showed significant effects of DSP-4 treatment (prefrontal cortex: *F*_1,15_ = 62.326, *p* < *0.001*, hippocampus: *F*_1,19_ = 7.188, *p = 0.016*) and surgery (prefrontal cortex: *F*_1,15_ = 25.013, *p* < *0.001*, hippocampus: *F*_1,19_ = 12.775, *p = 0.003*), as well as the interaction between treatment and surgery (prefrontal cortex: *F*_1,15_ = 24.860, *p* < *0.001*, hippocampus: *F*_1,19_ = 15.029, *p = 0.001*). Tukey’s *post hoc* test showed that both DSP-4 administration (prefrontal cortex: *p* < *0.001*, hippocampus: *p = 0.009*, Figure 10) and surgery (prefrontal cortex: *p = 0.006*, hippocampus: *p = 0.011*, Figure 10) reduced the levels of BDNF in the prefrontal cortex and hippocampus, while DSP-4 treatment had no significant effects on BDNF levels compared to the V + S group (prefrontal cortex: *p = 0.535*, hippocampus: *p = 0.968*, Figure 10).

## Discussion

The aim of our study was to evaluate the effects of LCNE on surgery-induced neuroinflammation and cognitive decline in rats. We demonstrated that DSP-4, a neurotoxic drug that selectively degenerates noradrenergic neurons originating from the LC, attenuated neuroinflammation in the prefrontal cortex and hippocampus, including decreased levels of proinflammatory cytokines, inhibited the activation of microglia and astrocytes, and improved hippocampal-dependent learning and memory. To our knowledge, this is the first report of the impacts of LCNE on neuroinflammation and cognitive decline in rats with POCD.

A number of studies have shown that neuroinflammation plays a key role in POCD. Peripheral aseptic inflammation is considered to be the beginning of neuroinflammation (Hoogland et al., 2015; Dokalis and Prinz, 2019; Subramaniyan and Terrando, 2019). Sterile surgery leads to the release of damage-associated molecular patterns (DAMPs) followed by the recruitment of leukocytes and the release of inflammatory cytokines (Medzhitov, 2008; McDonald and Kubes, 2011; Zindel and Kubes, 2020). Acute inflammation is necessary to clear the damaged tissue and engage tissue repair; however, an imbalanced immune response can become overwhelming and lead to a “cytokine storm” (Huber-Lang et al., 2018). Overexpressed cytokines cross the BBB, leading to neuroinflammation and a decline in cognition (Terrando et al., 2011).

In the surgery-induced POCD model (Hovens et al., 2014), plasma IL-6 levels were increased significantly the first 12 h after the surgery and decreased to baseline concentrations after 48 h, and the peak concentration occurred at 6 h postoperatively. In addition, the concentration of IL-1β in the prefrontal cortex and hippocampus significantly increased at 1 week postoperatively and returned to a level similar to or even lower than that of the control group at 2 and 3 weeks after the surgery. Our results demonstrated that in the early stage of inflammation, DSP-4 treatment increased surgery-induced systemic inflammation accompanied by an increased level of plasma IL-6. At 1 week postoperatively, the concentrations of plasma IL-6 returned to baseline; however, the level of plasma IL-1β showed that LCNE lesions had an effect on decreasing the inflammation induced by surgery. DSP-4 pretreatment inhibited systemic inflammation, accompanied by suppressed neuroinflammation, which included decreased levels of IL-6 and IL-1β and the reduced activation of microglia and astrocytes in the prefrontal cortex and hippocampus. Therefore, our research suggests that LCNE lesions mainly play an important anti-inflammatory role in rats with POCD.

These findings are consistent with the proinflammatory roles of LCNE in other disease models associated with neuroinflammation, such as acute and chronic stress (Johnson et al., 2005; Barnard et al., 2019) and Escherichia *coli*-induced septic encephalopathy (Johnson et al., 2008). Pretreatment with DSP-4 blocked the chronic stress-induced elevation in IL-1β in the hippocampus and attenuated the IL-1β increase in circulation (Johnson et al., 2005). In rat peripheral *E. coli* challenge, prior to the central administration of propranolol (a nonselective β receptor blocker) greatly attenuated the *E. coli*-induced increases in IL-1β levels in the brain, tissue, and plasma (Johnson et al., 2008).

These results confirmed that LCNE lesions were beneficial for reducing neuroinflammation, and several studies indicated that the activation of β-AR plays a key role in the effects of NE on neuroinflammation. Sugama et al. (2019) found that acute stress contributes to the activation of microglia in the hypothalamus, hippocampus, and thalamus, and an excitation was substantially inhibited by the β-blocker propranolol but was further activated by pretreatment with the α_2_ adrenergic receptor (α_2_-AR) blocker yohimbine. In the RAW 264.7 murine macrophage cell line, the activation of β_2_-AR leads to IL-1β and IL-6 production through ERK1/2- and p38-dependent activation of ATF-1 and ATF-2 transcription factors, thus playing a proinflammatory role (Tan et al., 2007). However, several studies have indicated that LCNE has an anti-inflammatory role in neuroinflammation. In mice of parkinson’s disease, the lesions of LCNE increased the inflammatory activity of microglia and reduced neurotrophic function (Yao et al., 2015). In a study on rat functional pain, continued activation of β-AR resulted in increased levels of TNF-α, IL-6, and IL-1β in CSF and activated microglia and astrocytes in the spinal cord (Zhang et al., 2018). The discrepancy in the effects of LCNE on neuroinflammation may be due to the different animal models and states of the receptors.

The physiological effects of NE are mediated by the three families of G-protein- coupled receptors, α_1_, α_2_, and β, each consisting of several subtypes. It is worth mentioning that β_2_-AR has been proven to be the key to NE regulating inflammation, and NE suppresses TLR-induced pro-inflammatory cytokine TNF-α secretion through β_2_-AR (Ağaç et al., 2018). Zhang et al. (2018) found that sustained stimulation of β_2_ and β_3_ receptors increased the levels of TNF-α, IL-6, and IL-1β in the plasma and CSF, while microglia and astrocytes in the spinal cord were activated. A recent study suggested that α_2_-AR also participates in regulating neuroinflammation, and dexmedetomidine treatment prevents LPS-induced cognitive decline and neuroinflammation by inhibiting nuclear factor kappa B (NF-κB) through a pathway mediated by α_2_-AR (Li et al., 2020). However, the specific pathophysiological mechanism by which LCNE regulates neuroinflammation is still unclear. Our results showed that under physiological conditions, the depletion of LCNE promoted the release of the central cytokines IL-6 and IL-1β and activated microglia and astrocytes in the prefrontal cortex and hippocampus. In contrast, in pathological settings, LCNE lesions have anti-inflammatory effects. The specific mechanism and reasons remain to be further explored, which may be related to the activation or suppression state of β receptors, and the effects of α_2_ receptors should not be overlooked.

The prefrontal cortex in rodents has been demonstrated to participate in advanced functions such as working memory, rule representation, response control, attention, and strategy shifting (Carlen, 2017). Our ELISA and immunofluorescence results suggested that the prefrontal cortex was affected by neuroinflammation with the release of cytokines (IL-6 and IL-1β) and the activation of microglia and astrocytes. However, behavioral outcomes associated with PFC, including NO recognition and MWM reversal tests, showed no significant change. The literature indicates that hippocampus-dependent learning and memory are especially vulnerable to inflammatory insults (Yirmiya and Goshen, 2011; Hovens et al., 2014). This finding may explain why, despite the increased neuroinflammation in the PFC, only hippocampal-dependent functions were impaired in our rats.

Brain-derived neurotrophic factor is involved in plasticity, neuronal survival, the formation of new synapses, and the modulation of excitatory and inhibitory neurotransmitter profiles (Edelmann et al., 2014; Panja and Bramham, 2014; Lima Giacobbo et al., 2019). It has been confirmed that BDNF plays a central role in forms of long-lasting synaptic plasticity associated with the consolidation of hippocampus-dependent memory (Botterill et al., 2015; Patterson, 2015; El Hayek et al., 2019). Referring to a previous study, surgery induced a decreasing concentration of BDNF in the hippocampus 2 weeks after the surgery, and the time point of minimum concentration was 3 weeks postoperatively (Hovens et al., 2014). We measured the levels of BDNF in the prefrontal cortex and hippocampus 3 weeks postoperatively, and the results showed that surgery and DSP-4 treatment downregulated the expression of BDNF when spatial learning and memory returned to baseline levels. Hypothetically, when BDNF was reduced for a long period, a compensatory mechanism was created to protect spatial memory function. Carretón et al. (2012) demonstrated the upregulation of TrkB expression in the hippocampus of BDNF^+/–^ mice, which can partially improve spatial learning and memory performance. This finding suggested that there was a compensatory mechanism to protect cognitive function after prolonged low levels of BDNF.

According to previous research, intraperitoneal injection of 50 mg/kg DSP-4 caused the degeneration of LCNE, which lasted more than 10 weeks (Archer et al., 1984). Chan’s results showed that 250 μg of DSP-4 intracerebroventricular injection was effective in depleting central NE (Chan et al., 1991). DSP-4 is capable of crossing the BBB and does not significantly influence BBB permeability (Tengvar et al., 1989). Our HPLC results suggested that intracerebroventricular injection of DSP-4 at a single dose of 400 μg per rat reduced the release of NE in the plasma, the PFC, and the hippocampus. DSP-4 treatment without surgery damaged hippocampal performance and increased systemic and central inflammation.

There are several limitations to our research. First, we just focused on the effect of LCNE degeneration on regulating systemic and central inflammation, not the receptors or signaling pathways leading to this phenomenon. Further study on modulating the inflammatory mechanism of DSP-4 will give us more ideas for preventing and improving POCD. Second, although DSP-4 is highly selective for LCNE neurons, it can produce minor depleting effects on serotonin (5-HT) (Jonsson et al., 1981). In this experiment, we did not design an indicator to judge the effect of DSP-4 on 5-HT. Nevertheless, the results of previous studies indicated that intraperitoneal injection of DSP-4 at 50 mg/kg does not significantly decrease the level of 5-HT in the CNS (Jonsson et al., 1981; Scullion et al., 2009). Third, we detected Iba-1 densitometry to assess the reactive states of microglia in the prefrontal cortex and hippocampus. Previous research has shown that microglia can produce cytotoxic or neuroprotective effects depending on the phenotypes activated (Tang and Le, 2016; Radandish et al., 2021; Rahimian et al., 2021). Our research evaluated the activation of microglia as a whole but did not distinguish between M1 and M2 phenotypes. Fourth, there might be learning effects due to the repeated behavioral tests. However, on one hand, we believe that the setting up of a vehicle group can counteract the learning effects; on the other hand, after a rest period (generally at least 1 week), by changing the platform location, both learning and relearning experiments can be accomplished (Terry, 2009). In our study, the time interval was 1 week, and the locations of objections in the NO/NL test and a platform in the MWM test were changed at different time points, to reduce possible learning effects. Fifth, POCD is the most common postoperative complication in elderly rats, and we used adult male rats because elderly rats are not readily available. Moreover, the study of Barnard et al. (2019) found that the reactions regulating brain IL-1β using the norepinephrine-β-AR pathway in male and female rats were diverse. Therefore, future investigations should include a comparison of the effects of DSP-4 treatment on neuroinflammation and behavioral changes in rats of different ages and sexes.

In conclusion, our results demonstrated that LCNE lesions increased peripheral inflammation in the early stage of inflammation and decreased neuroinflammation in the middle and advanced stage of inflammation, contributing to the improvement of cognitive function in rats with POCD. Thus, the LCNE system may be a potential therapeutic target for the treatment of POCD, pending further investigation.

## Data Availability Statement

The raw data supporting the conclusions of this article will be made available by the authors, without undue reservation.

## Ethics Statement

The animal study was reviewed and approved by The Institutional Animal Care and Use Committee (IACUC) at Practitioner Training institute of Hubei Province.

## Author Contributions

JW designed and performed the experiment, collected and analyzed the data, and prepared the manuscript. YZ was involved in preparing the animal models and participated in interpreting the results. KL contributed to behavioral testing. XL was involved in biochemical analysis. MG participated in the statistical analysis. MP contributed to the study concept and design, secured funding for the project, and prepared and critically revised the manuscript. All authors reviewed the manuscript.

## Conflict of Interest

The authors declare that the research was conducted in the absence of any commercial or financial relationships that could be construed as a potential conflict of interest.

## Publisher’s Note

All claims expressed in this article are solely those of the authors and do not necessarily represent those of their affiliated organizations, or those of the publisher, the editors and the reviewers. Any product that may be evaluated in this article, or claim that may be made by its manufacturer, is not guaranteed or endorsed by the publisher.
